# Sex difference in the association among nutrition, muscle mass, and strength in peritoneal dialysis patients

**DOI:** 10.1038/s41598-022-22722-y

**Published:** 2022-10-25

**Authors:** Jun Young Do, Seok Hui Kang

**Affiliations:** Division of Nephrology, Department of Internal Medicine, College of Medicine, Yeungnam University, 170 Hyeonchung-Ro, Nam-Gu, Daegu, 42415 Republic of Korea

**Keywords:** Diseases, Nephrology

## Abstract

Many peritoneal dialysis (PD) patients are malnourished, which leads to weakening owing to a decrease in muscle mass. However, this straightforward association could differ based on the sex of individuals. Further, studies on the sex-based association among nutrition, muscle mass, and strength would be helpful in choosing optimal interventions to improve the strength of patients on dialysis. The study aimed to assess the association between these three variables using mediation analysis. This retrospective study included prevalent PD patients (n = 199). Mediation analysis was conducted to investigate the effect of the appendicular lean mass (ALM) index on the association between the geriatric nutritional risk index (GNRI) and handgrip strength (HGS). The numbers of PD patients with a low ALM index, low HGS, or low GNRI score were 121 (60.8%), 109 (54.8%), and 70 (35.2%), respectively. The proportion of patients with a low ALM index was lower in females than in males, and those with a low HGS were less in patients without diabetes than that in those with diabetes. Patients ≥ 65 years old had a higher proportion of low ALM index or HGS than those < 65 years old. Low HGS was significantly associated with mortality in both sexes. In males, the HGS was correlated with the GNRI and ALM index; however, in females, partial correlation did not demonstrate an association among the GNRI, ALM index, and HGS. Mediation analysis showed that, in males, the GNRI was associated with HGS, and the association was partially mediated through the ALM index. These data reveal that the ALM index accounts for some, but not all, of the relationship between GNRI and HGS. There is not only a significant relationship between the ALM index and HGS, but also some direct relationship between GNRI and HGS. In females, there was no significant association between the GNRI or ALM index and HGS. These suggest that, for both sexes, nutritional supplementation and exercise should be recommended as the primary intervention for improving strength; however, other interventions for improving muscle quality could be considered as alternatives for maintaining strength in women undergoing PD.

## Introduction

Chronic kidney disease is a global health problem and the United States (US) Renal Data System has revealed that its prevalence is 14.9% in US adults^1^. Chronic kidney disease can progress to kidney failure with renal replacement therapy (KFRT), which requires renal replacement therapy for maintenance of life^2^. The prevalence of KFRT persistently increased owing to the effects of chronic diseases, environmental changes, and aging^3,4^. Peritoneal dialysis (PD) is an important modality among renal replacement therapies that are required by patients with KFRT. The numbers of prevalent patients undergoing PD are approximately 5960 and 58,636 in South Korea and USA, respectively^1,5^.

Patients undergoing PD are prone to pathologic conditions, including uremia, volume overload, acidosis, or bio-incompatible dialysate, which can result in increased energy expenditure, chronic inflammation, and multiple endocrine disorders^6^. These factors are associated with increased catabolism and decreased energy intake, which cumulatively lead to protein energy wasting^7^. This is associated with decreased muscle mass and/or strength, and consequently, with the development of physical disability^6,7^. Previous studies have demonstrated the importance of muscle strength, rather than muscle mass, as a functional indicator^8–10^. Furthermore, identification of factors related to strength is crucial for the prevention and treatment of physical disability.

Handgrip strength (HGS) is one of the most important prognostic factors for mortality in older patients or patients with chronic diseases^11–13^. The HGS is associated with muscle mass and quality. Muscle mass consists mainly of protein, and protein energy wasting can lead to a decrease in muscle mass. These results revealed that protein energy wasting can lead to a decrease in muscle mass, which results in low strength. However, previous studies have shown that associations among these three variables are not uniform and that sex differences exist^11–13^. A study using an adult population showed that serum albumin was positively associated with muscle mass in men but inversely associated with muscle mass in women^11^. Hayashida et al. evaluated a group of older individuals and showed that the positive association between muscle mass and strength is uniformly maintained in men but not in women^12^. A study of hospitalized patients showed that muscle mass is positively associated with strength in men but not in women^13^. These findings revealed that the associations among these variables should be analyzed separately according to sex. Furthermore, a few studies have investigated the sex difference in the association between the three variables in PD patients despite the high prevalence of low HGS or sarcopenia in the group. Further studies on the sex-based associations among these three variables would be helpful in choosing optimal interventions, beyond nutritional supplementation and exercise, for improving strength in patients with PD. Furthermore, data derived from these studies would be beneficial in planning future investigations regarding optimal treatment by sex and useful in identifying whether additional measurements for strength as a final outcome are needed. Therefore, this study aimed to assess the association among these three variables using mediation analysis.

## Methods

### Study population

We analyzed the data from a previous cross-sectional study that was conducted from September 2017 to November 2020^14^. Briefly, the study was conducted in a tertiary medical center on 214 prevalent patients undergoing PD between September 2017 and November 2020 (n = 123 in males and n = 91 in females), among whom 15 were excluded owing to insufficient data (*n* = 9) or having an amputated limb (*n* = 6). Consequently, 199 patients who undergone PD were included in this study (113 men and 86 women). HGS, body composition measurements, and laboratory test results were evaluated for all patients undergoing PD during the study period, and all measurements were performed on the same day of the peritoneal equilibration test. One trained nurse performed all measurements during the study period. The end point of the follow-up measurements was December 2021. The study was approved by the institutional review board (IRB) of Yeungnam University Medical Center (approval no: 2020-06-002). Informed consent was not obtained from the patients since the records and information of the participants were anonymized and de-identified prior to the analysis. The IRB also waived the need for obtaining informed consent. The study was conducted ethically in accordance with the World Medical Association Declaration of Helsinki. All clinical assessments were performed during routine patient care, and recorded using an electronic medical chart. The original data set was individually collected by researchers after IRB approval of the study protocol. All clinical data, including body composition, were manually collected using medical chart review, regardless of specific or standardized technique employed. Conversely, all personal information was de-identified after the collection of the relevant data.

### Variables

Baseline data on the age, sex, presence and type of diabetes mellitus (DM), dialysis modality, dialysis vintage (months), body mass index (kg/m^2^), weekly Kt/V_urea_, C-reactive protein level (mg/dL), 4-h dialysate-to-plasma creatinine concentration ratio (DP4Cr), urine volume (ml/day), edema index, and serum calcium (mg/dL), phosphorus (mg/dL), sodium (mEq/L), potassium (mEq/L), intact parathyroid hormone (i-PTH; pg/mL), and albumin (g/dL) levels were collected. All data were collected on the day of the modified 4.25% peritoneal equilibrium test, which is performed annually at our center. DM was defined based on a patient-reported history and medical record of DM diagnosis or medication. Dialysis modality was classified as continuous ambulatory PD or automated PD at the time of the test. Body mass index was calculated as the body weight per height squared.

Weekly Kt/V_urea_ was calculated using 24-h urine and dialysate as previously described^15^. Weekly Kt/V_urea_ was defined as 7 × (renal Kt + peritoneal Kt)/V_urea_. Renal Kt and peritoneal Kt were 24-h dialysate urea nitrogen/serum urea nitrogen and 24-h urine urea nitrogen/serum urea nitrogen, respectively. V_urea_ was calculated using Watson’s formula. DP4Cr was evaluated using a modified 4.25% peritoneal equilibration test, and the level was calculated using the creatinine level of the drained dialysate 4 h after injection, as per the blood creatinine level. Urine was collected for 24-h on the day preceding the peritoneal equilibrium test, and the total volume was recorded. The residual renal function (RRF, ml/min/1.73 m^2^) was calculated using creatinine and urea nitrogen excretion from 24-h urine collection as previously defined^16^. We also collected data for the peritoneal ultrafiltration volume and total output including the urine and peritoneal ultrafiltration volume.

The volume status was evaluated using bioimpedance analysis (InBody 770, Seoul, Korea), and measurements were performed in the morning. The InBody Body Composition Analyzer 770 is a multi-frequency bioimpedance analysis using impedance at 1, 5, 50, 250, 500, and 1000 kHz. The participants wore only a light gown and the peritoneal dialysate was drained out. They stood barefoot on the platform with the soles of their feet on the electrodes. They then grasped the handles of the unit with their thumb and fingers to maintain direct contact with the electrodes. The machine measured the total body water and extracellular water content. The edema index was defined as the extracellular water/total body water ratio obtained using the bioimpedance measurements. Serum albumin level was measured using the bromocresol purple method.

### Nutritional status, body composition, and HGS

HGS was measured using a digital dynamometer (Takei 5401; Takei Scientific Instruments Co., Ltd., Niigata, Japan) using the protocol from the American Society of Hand Therapists^17^. Briefly, the patient remained in a sitting position and positioned the adducted shoulder without rotation, flexed the forearm to 90°, and extended the wrist between 0 and 30°. The patient gripped the dynamometer, with the examiner supporting the dynamometer. The dominant arm was defined as the arm used for writing^18^. Each patient performed three trials with the dominant hand with a 60 s rest period between each trial. The maximum force was recorded for 6 s in each test. The maximum strength of three trials was selected. If a participant was ambidextrous, we measured the HGS in both hands and recorded the maximum values. Only one study patient was ambidextrous.

Body compositions were measured using a dual-energy X-ray absorptiometry (DXA) system (Hologic, Madison, WI, United States). Bone mineral content, fat mass, and lean mass were measured with DXA. Lean mass includes the soft tissues, excluding the bone and fat. Although other soft tissues, such as the organs, are included in lean mass, skeletal muscle mass is a major constituent of lean mass beyond muscle mass. Current guidelines suggest the use of appendicular lean mass (ALM) index from DXA for predicting muscle mass in both the general population and in dialysis patients^19,20^. All participants were required to extend overnight fasting until measurements were obtained, and to avoid exercise, showers, and fluids for more than 3-h before measurement. The scans were performed in the supine position and analyzed using the Hologic Discovery Wi software (version 13.3). Calibration of the densitometer was checked daily using a manufacturer-supplied standard calibration block and passed at −1.5 to + 1.5% of the control limits. Intra-observer measurement were performed for the intraclass correlations of the appendicular lean, trunk lean, and total fat masses. Intraclass correlation coefficients between the two measurements of ALM and total fat masses were 0.999 (95% confidence interval [CI], 0.997–1.000; *P* < 0.001) and 0.999 (95% CI, 0.998–1.000; *P* < 0.001), respectively. All measurements were performed by a technician accordance with the manufacturer’s manual, and measurements between observers were not evaluated. ALM was estimated using the sum of the lean masses of both extremities, and the index values were defined as the value per height square.

The Geriatric Nutritional Risk Index (GNRI) was calculated from a previously described equation: GNRI = [14.89 × albumin (g/dL)] + [41.7 × (body weight/ideal body weight)] ^21^. Body weight was measured while the patient was wearing a light gown after peritoneal dialysate drainage. The ideal body weight was calculated using Lorentz equations: for men, ideal body weight = height (cm)–100–[(height-150)/4]; for women, ideal body weight = height–100–[(height–150)/2.5]. Low group for ALM index, HGS, or GNRI was defined based on previous studies^19,21^. Briefly, low group for ALM index or HGS was defined as values from Asian Working Group for Sarcopenia; ALM index, < 7.0 kg/m^2^ in men and < 5.4 kg/m^2^ in women; HGS, < 28 kg in men and < 18 kg in women^19^. Low GNRI was defined as < 92 according to a previous study^21^.

### Statistical analysis

The data were analyzed using the IBM SPSS software (version 25.0; IBM Corp., Armonk, NY, USA). Distributions of continuous variable were tested using Kolmogorov–Smirnov test. The categorical variables are expressed as counts (percentages), and continuous variables with normal distribution are expressed as means ± standard deviations, and median (interquartile range) is used for non-normal distribution. Comparison of continuous variables between the sexes was performed using Student’s t-test for those with a normal distribution and Mann–Whitney U-test for those without a normal distribution. The univariate correlations between continuous variables were assessed using Pearson’s correlation for those with a normal distribution and Spearman’s correlation for those without a normal distribution. Partial correlations were adjusted for age and DM. For linear regression analyses, the dependent variable was HGS and the independent variable was the GNRI or ALM index. Model 1 was adjusted for age, presence of DM, C-reactive protein, DP4Cr, and weekly Kt/V_urea_. Model 2 was adjusted using similar factors, but also included the GNRI and ALM index in the adjustment. A mediation analysis was conducted using Baron and Kenny’s regression approach to investigate the mediating effect of the ALM index on the association between GNRI and HGS ^22^. A stepwise approach was followed to ensure that the following four conditions were met: (a) an established association between the GNRI and the HGS, (b) an established relationship between the GNRI and ALM index, (c) an association between the ALM index and the HGS, and (d) after adjustment for the ALM index, an association between the GNRI and HGS that was no longer significant, or which changed for the standardized beta (St-β) value. The Kaplan–Meier analysis was used to plot survival curves among the groups, and the log-rank method was used to determine statistical significance. The level of statistical significance was set at *P* < 0.05.

## Results

### Participant clinical characteristics

The numbers of men and women were 113 (56.8%) and 86 (43.2%), respectively (Table 1). The mean age of the male and female patients was 55.4 ± 12.5 and 55.7 ± 12.0 years, respectively. The dialysis period was 49 (63) months. The proportions of patients with DM and those undergoing automated PD were greater in men than in women. Ninety-eight patients had DM, and 92 (93.9%) had type 2 DM. Body mass index, C-reactive protein, DP4Cr, and urine volume were greater in men than in women. The RRF values were 0 (1.9) ml/min/1.73 m^2^ in men and 0 (0.3) ml/min/1.73 m^2^ in female, and no significant difference was found in the RRF between the sexes (*P* = 0.831). The weekly Kt/V_urea_, and serum calcium levels were greater in women than in men. There were no significant differences in age, dialysis period, edema index, and i-PTH, phosphorus, sodium, potassium, and albumin levels between men and women. The peritoneal ultrafiltration volume was 905 ± 709 mL/day in men and 1015 ± 497 mL/day in women (*P* = 0.218). The total output was 1444 ± 654 mL/day in men and 1285 ± 536 mL/day in women (*P* = 0.068). Thus, no significant differences were found in peritoneal ultrafiltration volume and total output between men and women. All women except three patients were in menopause. The total fat mass index in men and women was 6.8 ± 2.3 and 7.8 ± 2.6 kg/m^2^, respectively (*P* = 0.005). Correlation analyses between GNRI, ALM index, HGS, dialysis vintage, weekly Kt/Vurea, C-reactive protein, DP4Cr, and urine volume were performed (Table S1). The analyses revealed that weekly Kt/Vurea, C-reactive protein, and DP4Cr were correlated with GNRI, ALM index, and HGS. Therefore, these indicators were added as covariates in the multivariate analyses.

**Table 1:** Participant clinical characteristics.

	Total (*n* = 199)	Men (*n* = 113)	Women (*n* = 86)	*P*
Age (years)	55.5 ± 12.2	55.4 ± 12.5	55.7 ± 12.0	0.865
Diabetes mellitus (%)	98 (49.5%)	66 (58.4%)	32 (37.2%)	0.003
Automated peritoneal dialysis	58 (29.0%)	39 (34.5%)	18 (20.9%)	0.036
Dialysis vintage (months)	49 (63)	43 (51)	58 (85)	0.056
Body mass index (kg/m^2^)	24.7 ± 3.8	25.3 ± 3.9	23.8 ± 3.4	0.004
Weekly Kt/V_urea_	1.92 ± 0.46	1.83 ± 0.51	2.04 ± 0.36	0.002
C-reactive protein (mg/dL)	0.18 (0.39)	0.19 (0.37)	0.13 (0.41)	0.028
DP4Cr	0.66 ± 0.13	0.68 ± 0.12	0.64 ± 0.15	0.037
Urine volume (ml/day)	50 (600)	50 (844)	50 (419)	0.018
Edema index	0.400 ± 0.013	0.399 ± 0.014	0.401 ± 0.011	0.222
Intact-parathyroid hormone (pg/mL)	280 (303)	280 (233)	284 (401)	0.185
Serum calcium (mg/dL)	8.3 ± 0.9	8.1 ± 0.9	8.6 ± 0.9	< 0.001
Serum phosphorus (mg/dL)	4.9 ± 1.4	4.9 ± 1.4	4.9 ± 1.3	0.936
Serum sodium (mEq/L)	136 ± 4	136 ± 4	136 ± 3	0.917
Serum potassium (mEq/L)	4.5 ± 0.7	4.5 ± 0.7	4.5 ± 0.7	0.979
Serum albumin (g/dL)	3.6 ± 0.5	3.6 ± 0.5	3.6 ± 0.4	0.995

Data are expressed as mean ± standard deviation for continuous variables with normal distribution, median (interquartile range) for continuous variables without normal distribution and as numbers (percentages) for categorical variables. P-values are tested using Student’s t-test for continuous variables with normal distribution, Mann–Whitney U test for continuous variables without normal distribution, and Pearson’s χ^2^ or Fisher’s exact tests for categorical variables. Abbreviations: DP4Cr, four-hour dialysate-to-plasma creatinine concentration ratio.

The numbers of PD patients with low ALM index, low HGS, and low GNRI were 121 (60.8%), 109 (54.8%), and 70 (35.2%), respectively. We also analyzed the data according to subgroups by age, sex, or the presence of DM. The percentages of low values in men and women were 68.1% and 51.2% for the ALM index (*P* = 0.015), 51.3% and 59.3% for the HGS (*P* = 0.263), and 31.9% and 39.5% for the GNRI (*P* = 0.261), respectively. The percentages of low values in patients without or with diabetes were 56.4% and 65.3% for the ALM index (*P* = 0.200), 45.5% and 64.3% for the HGS (*P* = 0.008), and 30.7% and 39.8% for the GNRI (*P* = 0.179), respectively. The percentages of low values in patients aged < 65 and ≥ 65 were 56.9% and 73.9% for the ALM index (*P* = 0.038), 47.7% and 78.3% for the HGS (*P* < 0.001), and 34.0% and 39.1% for the GNRI (*P* = 0.522), respectively. These results revealed that that proportion of patients with low ALM index were lower in women than in men and those with low HGS were lower in patients without DM than that in those with DM. Patients ≥ 65 years old had higher proportions of low ALM index or low HGS than those < 65 years old. There were no significant differences in the proportions of low GNRI according to sex, age, or the presence of DM.

**Figure 1:**
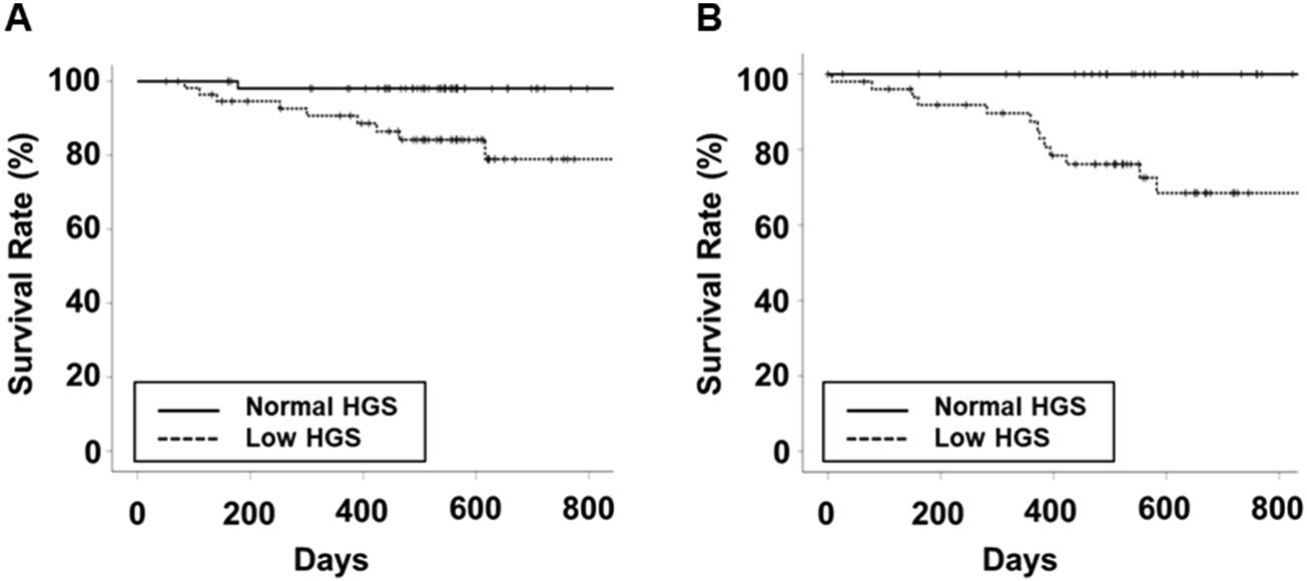
Kaplan–Meier curves of patient survival according to HGS for (**A**) men and (**B**) women. Abbreviations: HGS, handgrip strength.

The median follow-up duration in the male and female patients were 539 (191) and 548 (331) days, respectively. The survival rates in men with normal and low HGS were 98.1 and 94.6% at 200 days and 98.1 and 84.2% at 600 days, respectively (*P* = 0.009, Fig. 1). The survival rates in women with normal and low HGS were 100 and 91.9% at 200 days and 100 and 68.5% at 600 days, respectively (*P* < 0.001). Low HGS was significantly associated with mortality in both sexes.

### Correlation between GNRI, ALM index, and HGS

**Figure 2:**
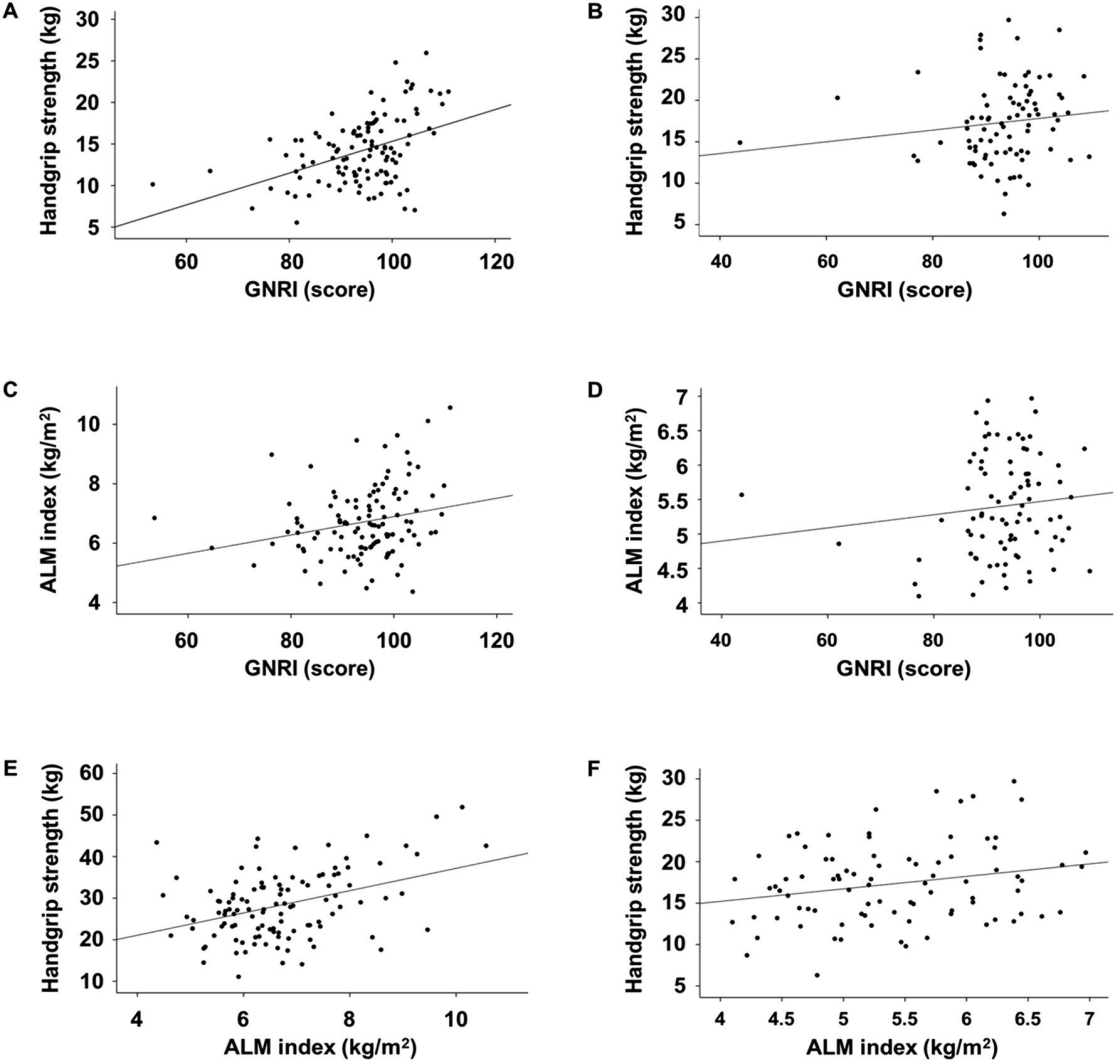
Scatter plots among GNRI, ALM index, and handgrip strength by sex. Handgrip strength and GNRI in men (**A**) and women (**B**). ALM index and GNRI in men (**C**) and women (**D**). Handgrip strength and ALM index in men (**E**) and women (**F**). Abbreviations: GNRI, Geriatric Nutritional Risk Index; ALM, appendicular lean mass.

The median GNRI and mean ALM index values were 95.8 (10.6) and 6.7 ± 1.2 kg/m^2^ in men, and 94.3 (8.9) and 5.5 ± 0.9 kg/m^2^ in women, respectively (*P* = 0.272 for GNRI and *P* < 0.001 for ALM index). The mean HGS in men and women was 28.4 ± 8.1 kg and 17.3 ± 4.8 kg, respectively (*P* < 0.001). In men, the HGS correlated with the GNRI and ALM index (Table 2 and Fig. 2); however, in women, partial correlation did not demonstrate an association between the ALM index and HGS.

**Table 2:** Correlation analyses among variables.

	Univariate correlation				Partial correlation			
	GNRI		ALM index		GNRI		ALM index	
	*r*	*P*	*r*	*P*	*r*	*P*	*r*	*P*
**Men**								
ALM index (kg/m^2^)	0.233	0.013	–	–	0.223	0.023	–	–
HGS (kg)	0.380	< 0.001	0.393	< 0.001	0.338	< 0.001	0.313	0.001
**Women**								
ALM index (kg/m^2^)	0.173	0.112	–	–	0.148	0.191	–	–
HGS (kg)	0.229	0.034	0.234	0.030	0.012	0.917	0.155	0.170

The data are expressed as correlation coefficients. *P* -values are tested using Pearson’s correlation for variables with normal distribution and Spearman correlation for those without normal distribution. Partial correlations are adjusted for age, the presence of diabetes mellitus, C-reactive protein, DP4Cr, and weekly Kt/V_urea_. Abbreviations: GNRI, Geriatric Nutritional Risk Index; ALM, appendicular lean mass; HGS, handgrip strength; DP4Cr, four-hour dialysate-to-plasma creatinine concentration ratio.

### Mediation analysis using the GNRI, ALM index, and HGS

**Figure 3:**
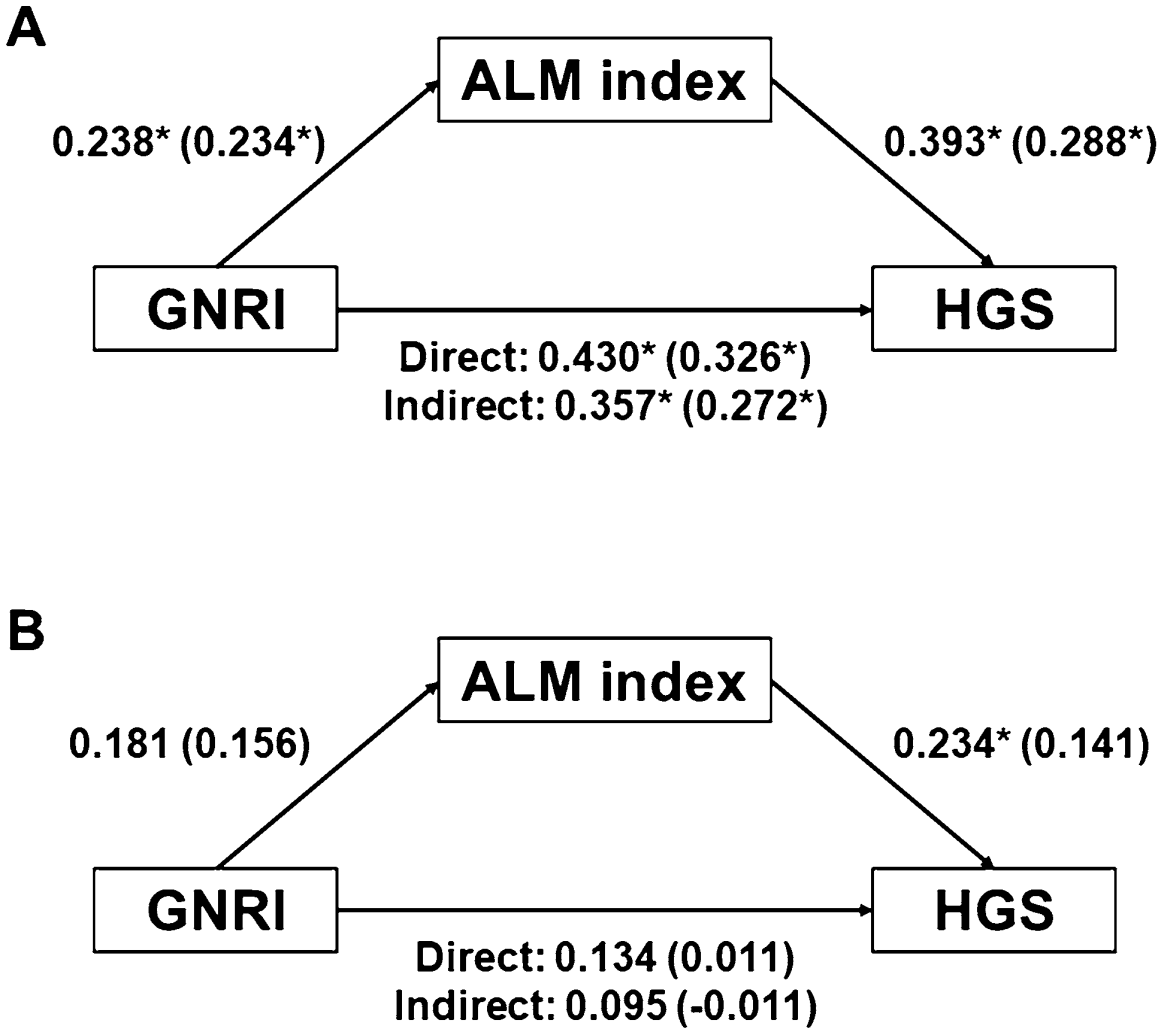
The relationship between GNRI, ALM index, and HGS in men (**A**) and in women (**B**). Numbers indicate standardized β value from univariate linear regression and numbers in bracket denote standardized β value from multivariate linear regression. Multivariate analyses are adjusted for age, the presence of diabetes mellitus, C-reactive protein, DP4Cr, weekly Kt/Vurea. Direct means linear regression using GNRI (independent variable) and HGS (dependent variable). Indirect indicates linear regression using GNRI (independent variable), ALM index (mediator), and HGS (dependent variable). *P < 0.05. Abbreviations: GNRI, Geriatric Nutritional Risk Index; ALM, appendicular lean mass; HGS, handgrip strength.

The Baron and Kenny analysis was used to determine whether the association between the GNRI and HGS was mediated via ALM index (Table 3). In men, GNRI (independent variable) was associated with ALM index (mediator) and HGS (dependent variable) (Fig. 3). ALM index is associated with HGS. Linear regression analyses using Model 2 revealed trends similar to those obtained using univariate analysis. Model 2 revealed that the association of the GNRI and HGS remained significant; however, the St-β value decreased.

**Table 3:** Linear regression analysis to assess the association between GNRI, ALM index, and HGS.

	Variables	St-β	*P* value	F	Adjusted R^2^
**Men**					
Univariate	GNRI	0.430	< 0.001	25.232	0.185
	ALM index	0.393	< 0.001	20.276	0.154
Model 1	GNRI	0.326	< 0.001	9.865	0.367
	ALM index	0.288	0.001	9.378	0.356
Model 2	GNRI	0.272	0.003	10.017	0.410
	ALM index	0.230	0.008		
**Women**					
Univariate	GNRI	0.134	0.218	1.540	0.018
	ALM index	0.234	0.030	4.866	0.055
Model 1	GNRI	0.011	0.917	3.547	0.214
	ALM index	0.141	0.170	3.951	0.233
Model 2	GNRI	–0.011	0.922	3.345	0.233
	ALM index	0.142	0.173		

HGS is considered as the dependent variable. Model 1 is adjusted for age, the presence of diabetes mellitus, C-reactive protein, DP4Cr, and weekly Kt/V_urea_. Model 2 is adjusted using similar factors but also includes GNRI and ALM index in the adjustment. Abbreviations: GNRI, geriatric nutritional risk index; St-β, standardized beta; ALM, appendicular lean mass; HGS, handgrip strength; DP4Cr, four-hour dialysate-to-plasma creatinine concentration ratio.

Univariate linear regression in women indicated that the ALM index (independent variables) exhibited a positive association with HGS. The association between the GNRI and ALM index was not significant. Model 1 and 2 demonstrated a negative association between HGS and GNRI and/or ALM index. These results show that the GNRI was associated with HGS, and that the association was partially mediated through the ALM index in men. In women, there was no significant association between the GNRI or ALM index and HGS.

### Subroup analyses according to sex

Tables S2, S3, and S4 show the data using the total cohort and the DM or Kt/V_urea_ groups (DM or non-DM, and low Kt/V_urea_ or high Kt/V_urea_). Subgroup analyses showed that the association among the three variables was greater in men than in women, regardless of DM. In addition, subgroup analyses showed that the association among the three variables was the greatest in men with low Kt/V_urea_. Although the association was weaker in men with high Kt/V_urea_ than in men with low Kt/V_urea_, associations were greater in men regardless of the Kt/V_urea_ group.

### Variables associated with ALM index

ALM index is not easily evaluated compared to the other variables. Therefore, we aimed to identify variables that could predict the ALM index, and performed univariate and multivariate linear regression analyses using the ALM index as the dependent variable. The results revealed that age and HGS were associated with the ALM index in men; however, no specific variables could predict the ALM index in women (Table 4). Furthermore, the odds ratio for low ALM index in men was 2.97 (95% CI, 1.30–6.80) for patients with low HGS compared to those without low HGS. The odds ratio for a low ALM index in women was 1.76 (95% CI, 0.74–4.19) for patients with low HGS compared to those without low HGS. This revealed that the HGS index was useful to predicting the ALM index in men, but not in women.

**Table 4:** Linear regression analysis to predict ALM index.

	Men				Women			
	Univariate		Multivariate		Univariate		Multivariate	
	St-β	*P*	St-β	*P*	St-β	*P*	St-β	*P*
Age	−0.365	0.000	−0.228	0.027	−0.165	0.129	−0.082	0.497
DM	−0.008	0.930	−0.154	0.108	−0.030	0.783	−0.042	0.714
CRP	−0.081	0.402	0.028	0.764	−0.135	0.219	−0.052	0.662
Weekly Kt/V_urea_	−0.057	0.551	−0.049	0.585	0.039	0.720	0.106	0.366
DP4Cr	0.101	0.289	0.110	0.235	−0.001	0.993	−0.008	0.941
GNRI	0.238	0.011	0.139	0.188	0.181	0.095	0.155	0.194
HGS	0.393	0.000	0.293	0.008	0.234	0.030	0.168	0.173

The ALM index is used as the dependent variable. Multivariate analyses are adjusted for GNRI, HGS, and variables from Model 1. Abbreviations: ALM, appendicular lean mass; St-β, standardized beta; DM, diabetes mellitus; CRP, C-reactive protein; DP4Cr, four-hour dialysate-to-plasma creatinine concentration ratio; GNRI, geriatric nutritional risk index; HGS, handgrip strength.

## Discussion

Our study investigated the association between the GNRI or ALM index and HGS in patients with stable PD. Our results show that in men, GNRI scores were associated with HGS, and that the association was partially mediated through the ALM index. In women, there was no significant association between the GNRI or ALM index and HGS.

Adequate nutritional status is associated with the maintenance of muscle mass, which is helpful in maintaining muscle strength. These associations are well-known causal relationships that have been demonstrated in studies involving various populations^23–26^. We evaluated the GNRI as a nutritional indicator and the ALM index using DXA for muscle mass measurement. Previous studies have revealed that the GNRI is an important nutritional index associated with morbidities or mortality in patients undergoing PD^27,28^. DXA is recommended as a useful method for predicting muscle mass measurements in patients undergoing PD despite its inherent limitation in bias by volume status^20^. Our study showed that, in men, the GNRI as a nutritional index is associated with both the ALM index and HGS; however, the effect of HGS was partially mediated by the ALM index. This indicated that nutritional status would influence muscle strength via other effects, regardless of the muscle mass. Although our study did not evaluate factors other than muscle mass, further studies are warranted to identify the other factors that influence muscle strength.

Our study did not demonstrate an association between the GNRI or ALM index and HGS in women. Muscle strength is associated with both neural and muscular factors; however, differences exist in the extent of their influence according to sex. The high testosterone level in men is associated with greater muscle mass in men than that in women, and the high estrogen level in women is associated with an increase in the bone mass to muscle mass^29–32^. In men, muscle mass correlates with strength; however, in women, strength more closely correlates with other factors, such as neural factors or change in muscle quality, owing to their limited muscle volume^12,29^. In our study, all female patients were menopausal, except for three. The inclusion of menopause in multivariate analyses would have been difficult because of an imbalance of the proportion of patients in menopause compared to those not. Although our study did not include data on intramuscular fat, the total fat mass index was higher in women than in men. A previous study assessing hemodialysis patients revealed that relative intramuscular fat was greater in women than in men^33^.

Dialysis patients were prone to uremic conditions, which are associated with insulin resistance and neuropathy^34^. These changes influence the muscle quality in dialysis patients. Therefore, dialysis patients have decreased muscle strength despite appropriate muscle mass compared with the general population. Previous studies on general and older populations have demonstrated a positive association between muscle mass and strength, but the association between the two variables was weaker in women than in men^12,35^. In addition, the increase in muscle mass due to nutritional supplements was stronger in men than in women^36^. These findings reveal that other factors, such as muscle quality or neuromuscular interaction, may also be important for strength in women in addition to nutritional status or muscle mass. Considering two issues, i.e., the association between muscle quality and uremic condition and the association between strength and muscle quality in women, changes in muscle quality in dialysis patients would be associated with more prominent changes in strength in female dialysis patients than in male dialysis patients. These findings reveal that the discrepancy in the association between muscle strength and muscle mass or nutritional status according to sex is greater than that in the general population.

Although the correlation among ALM index, GNRI, and HGS was significant, the values ranged from 0.2 to 0.4, which suggest weak correlations. The lack of a significant association in women might have been because of the small number of female patients. The correlation coefficients between the variables were small, and these low values may be associated with other confounding factors associated with muscle mass, strength, and nutritional status, or the small sample size. However, we aimed to focus on the sex-based difference among the three variables, as shown in Fig. 2. These plots reveal definite sex differences in trends among the three variables, despite having small correlation coefficients. Moreover, Pearson’s, Spearman, or partial correlation coefficients between the GNRI, ALM index, and HGS were persistently greater in men than in women.

Our results could be helpful in the selection of appropriate interventions to maintain muscle strength in patients undergoing PD. Nutritional supplementation plays a major role in maintaining muscle mass and strength. Furthermore, previous studies have shown that resistance exercise increases both muscle mass and strength^37^. Our results revealed a sex-based difference in the association between the three variables, with a greater association between strength and nutritional status or muscle mass in men than that in women. Therefore, current management options using nutritional supplementation and exercise are more efficient in men than in women, and additional interventions for improving muscle quality may be more efficient in women than in men. However, our results should not be interpreted as showing the lack of importance for interventions using nutritional supplementation and exercise in women. For both sexes, nutritional supplementation and exercise should be recommended as first-line intervention for improving strength. Other interventions, such as improvement in muscle quality through the treatment of insulin resistance or increased muscle-nerve contact through exercise training, could be considered as alternatives for maintaining strength in females undergoing PD. On the other hand, non-association between these three variables in women may lead to the requirement for additional measurements for strength. Our study revealed an association between HGS and patient survival, which justifies our evaluation of the factors associated with HGS. Both the ALM index and HGS are well-known indicators for predicting muscle quantity or quality, but the association between the two variables differed according to sex. In men, the ALM index was associated with age and HGS. However, in women, the ALM index did not predict HGS, which indicates the requirement for additional measurements for strength for this group. Strength and muscle mass did not have the same clinical significance. However, considering the convenience and low cost of HGS measurement and its association with prognosis, clinicians may consider additional measurements of HGS in PD patients, especially in women, which measure beyond just the muscle mass or nutritional status.

In our study, the weekly Kt/V_urea_ was greater in women than in men. Women have a smaller volume of urea distribution (V_urea_) than men, which is associated with greater weekly Kt/V_urea_ in women than in men. Debowska et al. evaluated the relationship between dialysis adequacy and body composition and showed that weekly Kt/V_urea_ was strongly influenced by body size^38^. In addition, dialysis adequacy is associated with inflammation in PD patients, and higher dialysis adequacy in women may lead to lower CRP levels than in men^39^.

Our study had certain limitations. First, this was a single-center, cross-sectional, retrospective study. Our design did not identify a definite causal relationship between the variables. Moreover, our study sample size was insufficient to adjust for all possible confounding factors, and could have influenced the non-significant associations between the GNRI or ALM index and HGS in women. However, it is difficult to perform a prospective study with a large sample size in patients with a low prevalence conditions, such as PD. The preliminary results from this study may be useful in driving further investigations to draw more robust conclusions. Second, our study had certain methodological drawbacks. Our study enrolled PD patients alone, which is an inherent limitation of our study. Further studies including healthy populations, patients with non-renal disease, and those undergoing PD would be useful in identifying independent associations among the nutritional index, muscle mass, and strength based on sex and/or dialysis effect. In addition, the ALM index was evaluated using DXA; however, muscle mass measurements using DXA are influenced by the volume status. Nevertheless, the recent Kidney Disease Outcomes Quality Initiative guidelines recommend the use of DXA for the measurement of muscle mass in patients undergoing dialysis, despite the limitation by volume^20^.

## Conclusions

The present study demonstrated that, in men, the GNRI was associated with both the ALM index and HGS, and the association between the GNRI and HGS was partially mediated by the ALM index. However, no such association was found between these variables in women. These suggest that, for both sexes, nutritional supplementation and exercise should be recommended as a primary intervention for improving strength, but other interventions for improvement in muscle quality should be considered as alternatives for maintaining strength in women undergoing PD. Considering the limitations of our study, prospective studies using a larger number of patients including the general population and patients with dialysis or other chronic disease are needed to identify a definitive causal-relationship between these indicators for optimal sex-based interventions.

## Supplementary Information

Supplementary Information 1.

Supplementary Information 2.

Supplementary Information 3.

Supplementary Information 4.

## Data Availability

All data generated or analyzed during this study are included in the published article.
