# Supplementary material for: Sex difference in the association among nutrition, muscle mass, and strength in peritoneal dialysis patients
Source: Sci Rep. 2022 Oct 25;12:17900. doi: 10.1038/s41598-022-22722-y (PMC9596441; doi:10.1038/s41598-022-22722-y)
Supplement: Supplementary file 1 — Supplementary Information 1. [file 41598_2022_22722_MOESM1_ESM.docx]

**Table S1. Correlation analysis among variables**

|  | **Men** | | | | | |  | **Women** | | | | | | | | |  |
| --- | --- | --- | --- | --- | --- | --- | --- | --- | --- | --- | --- | --- | --- | --- | --- | --- | --- |
|  | **GNRI** | | **ALM index** | | **HGS** | |  | **GNRI** | | | | **ALM index** | | | **HGS** | |  |
|  | ***r*** | ***P*** | ***r*** | ***P*** | ***r*** | ***P*** |  | | ***r*** | ***P*** | ***r*** | | ***P*** | ***r*** | | ***P*** |  |
| Dialysis vintage | –0.031 | 0.746 | –0.137 | 0.148 | 0.004 | 0.968 |  | | 0.048 | 0.665 | 0.051 | | 0.644 | –0.128 | | 0.243 |  |
| Weekly Kt/V_urea_ | 0.126 | 0.187 | –0.057 | 0.551 | –0.032 | 0.735 |  | | –0.292 | 0.006 | 0.039 | | 0.720 | –0.160 | | 0.887 |  |
| C-reactive protein | –0.157 | 0.102 | –0.057 | 0.554 | –0.040 | 0.681 |  | | –0.171 | 0.118 | –0.215 | | 0.048 | –0.407 | | <0.001 |  |
| DP4Cr | –0.262 | 0.005 | 0.101 | 0.289 | –0.049 | 0.608 |  | | –0.085 | 0.437 | –0.001 | | 0.993 | –0.021 | | 0.850 |  |
| Urine volume | 0.184 | 0.052 | 0.104 | 0.275 | 0.135 | 0.156 |  | | –0.076 | 0.490 | –0.122 | | 0.262 | 0.128 | | 0.241 |  |

The data are expressed as correlation coefficients. *P*-values were tested using Pearson’s correlation for variables with normal distribution and Spearman correlation for those without normal distribution.

**Abbreviations**: GNRI, Geriatric Nutritional Risk Index; ALM, appendicular lean mass; HGS, handgrip strength; DP4Cr, four-hour dialysate-to-plasma creatinine concentration ratio
