# Supplementary material for: Sex difference in the association among nutrition, muscle mass, and strength in peritoneal dialysis patients
Source: Sci Rep. 2022 Oct 25;12:17900. doi: 10.1038/s41598-022-22722-y (PMC9596441; doi:10.1038/s41598-022-22722-y)
Supplement: Supplementary file 2 — Supplementary Information 2. [file 41598_2022_22722_MOESM2_ESM.docx]

**Table S2. Correlation analysis among variables according to various groups**

|  | **Pearson’s correlation** | | | | |  | | **Partial correlation** | | | | | |  |
| --- | --- | --- | --- | --- | --- | --- | --- | --- | --- | --- | --- | --- | --- | --- |
|  | **GNRI** | | | **ALM index** | |  | | **GNRI** | | | | **ALM index** | |  |
|  | ***r*** | ***P*** | ***r*** | | ***P*** |  | | | ***r*** | ***P*** | ***r*** | | ***P*** |  |
| Total cohort |  |  |  | |  | |  | |  |  |  | |  |  |
| ALM index (kg/m^2^) | 0.200 | 0.005 | – | | – | |  | | 0.168 | 0.022 | – | | – |  |
| HGS (kg) | 0.263 | <0.001 | 0.551 | | <0.001 | |  | | 0.241 | 0.001 | 0.275 | | <0.001 |  |

Data are expressed as correlation coefficients *P*-values were tested using Pearson’s correlation for variables with normal distribution and Spearman correlation for those without normal distribution. Partial correlation was adjusted for age, sex, presence of diabetes mellitus, C-reactive protein, DP4Cr, and weekly Kt/V_urea_.

**Abbreviations**: GNRI, geriatric nutritional risk index; ALM, appendicular lean mass; HGS, handgrip strength; DP4Cr, four-hour dialysate-to-plasma creatinine concentration ratio
