# Supplementary material for: Sex difference in the association among nutrition, muscle mass, and strength in peritoneal dialysis patients
Source: Sci Rep. 2022 Oct 25;12:17900. doi: 10.1038/s41598-022-22722-y (PMC9596441; doi:10.1038/s41598-022-22722-y)
Supplement: Supplementary file 3 — Supplementary Information 3. [file 41598_2022_22722_MOESM3_ESM.docx]

**Table S3. Correlation analysis among variables according to the presence of DM and sex.**

|  | **Pearson’s correlation** | | | | |  | | **Partial correlation** | | | | | |  |
| --- | --- | --- | --- | --- | --- | --- | --- | --- | --- | --- | --- | --- | --- | --- |
|  | **GNRI** | | | **ALM index** | |  | | **GNRI** | | | | **ALM index** | |  |
|  | ***r*** | ***P*** | ***r*** | | ***P*** |  | | | ***r*** | ***P*** | ***r*** | | ***P*** |  |
| Men without DM |  |  |  | |  | |  | |  |  |  | |  |  |
| ALM index (kg/m^2^) | 0.273 | 0.063 | – | | – | |  | | 0.248 | 0.118 | – | | – |  |
| HGS (kg) | 0.575 | <0.001 | 0.423 | | 0.003 | |  | | 0.536 | <0.001 | 0.306 | | 0.051 |  |
| Men with DM |  |  |  | |  | |  | |  |  |  | |  |  |
| ALM index (kg/m^2^) | 0.216 | 0.081 | – | | – | |  | | 0.191 | 0.144 | – | | – |  |
| HGS (kg) | 0.221 | 0.075 | 0.408 | | 0.001 | |  | | 0.144 | 0.272 | 0.316 | | 0.014 |  |
| Women without DM |  |  |  | |  | |  | |  |  |  | |  |  |
| ALM index (kg/m^2^) | 0.242 | 0.078 | – | | – | |  | | 0.111 | 0.448 | – | | – |  |
| HGS (kg) | 0.142 | 0.305 | 0.286 | | 0.036 | |  | | –0.033 | 0.821 | 0.180 | | 0.215 |  |
| Women with DM |  |  |  | |  | |  | |  |  |  | |  |  |
| ALM index (kg/m^2^) | –0.142 | 0.437 | – | | – | |  | | –0.171 | 0.384 | – | | – |  |
| HGS (kg) | 0.156 | 0.394 | 0.142 | | 0.439 | |  | | 0.077 | 0.696 | –0.006 | | 0.976 |  |

Data are expressed as correlation coefficients, and *P*-values were tested using Pearson’s correlation for variables with normal distribution and Spearman’s correlation for those without normal distribution. Partial correlation was adjusted for age, C-reactive protein level, DP4Cr level, and weekly Kt/V_urea_.

**Abbreviations**: ALM, appendicular lean mass; DM, diabetes mellitus; DP4Cr, four-hour dialysate-to-plasma creatinine concentration ratio; GNRI, geriatric nutritional risk index; HGS, handgrip strength.
