# Supplementary material for: Sex difference in the association among nutrition, muscle mass, and strength in peritoneal dialysis patients
Source: Sci Rep. 2022 Oct 25;12:17900. doi: 10.1038/s41598-022-22722-y (PMC9596441; doi:10.1038/s41598-022-22722-y)
Supplement: Supplementary file 4 — Supplementary Information 4. [file 41598_2022_22722_MOESM4_ESM.docx]

**Table S4. Correlation analysis among variables according to weekly Kt/V_urea_ and sex**

|  | **GNRI** | | **ALM index** | |  |
| --- | --- | --- | --- | --- | --- |
|  | ***r*** | ***P*** | ***r*** | ***P*** |  |
| Men with low Kt/V_urea_ (n = 57) |  |  |  |  |  |
| ALM index (kg/m^2^) | 0.281 | 0.034 | – | – |  |
| HGS (kg) | 0.488 | <0.001 | 0.556 | <0.001 |  |
| Men with high Kt/V_urea_ (n = 56) |  |  |  |  |  |
| ALM index (kg/m^2^) | 0.187 | 0.171 | – | – |  |
| HGS (kg) | 0.366 | 0.006 | 0.199 | 0.145 |  |
| Women with low Kt/V_urea_ (n = 13) |  |  |  |  |  |
| ALM index (kg/m^2^) | 0.464 | 0.110 | – | – |  |
| HGS (kg) | 0.333 | 0.267 | 0.527 | 0.064 |  |
| Women with high Kt/V_urea_ (n = 73) |  |  |  |  |  |
| ALM index (kg/m^2^) | 0.066 | 0.579 | – | – |  |
| HGS (kg) | 0.107 | 0.365 | 0.176 | 0.136 |  |

Data are expressed as correlation coefficients, and *P*-values were tested using Pearson’s correlation for variables with normal distribution and Spearman’s correlation for those without normal distribution. Low Kt/V_urea_ and high Kt/V_urea_ were defined as < 1.7 and ≥ 1.7, respectively.

**Abbreviations**: ALM, appendicular lean mass; DP4Cr, four-hour dialysate-to-plasma creatinine concentration ratio; GNRI, geriatric nutritional risk index; HGS, handgrip strength.
